# Supplementary material for: Usefulness of Orientation to the Year as an Aid to Case Finding of Mild Cognitive Impairment or Depression in Community-Dwelling Older Adults
Source: Int J Environ Res Public Health. 2021 Jul 30;18(15):8096. doi: 10.3390/ijerph18158096 (PMC8345456; doi:10.3390/ijerph18158096)
Supplement: Supplementary file 1 [file ijerph-18-08096-s001.zip › Table S1.pdf]

Table S1. Number of errors in three-item recall (tree, car, hat) tests for the diagnosis of MCI

Number of errors	Sensitivity	Specificity	PPV	NPV	Accuracy	Youden's index
1	69.9%	40.5%	25.1%	82.5%		0.104
2	37.8%	75.0%	30.2%	80.9%	66.8%	0.129
3	18.1%	90.8%	35.9%	79.5%		0.089

PPV, positive predictive value; NPV, negative predictive value; Accuracy, proportion of true results among the total number of cases examined
