# Supplementary material for: Usefulness of Orientation to the Year as an Aid to Case Finding of Mild Cognitive Impairment or Depression in Community-Dwelling Older Adults
Source: Int J Environ Res Public Health. 2021 Jul 30;18(15):8096. doi: 10.3390/ijerph18158096 (PMC8345456; doi:10.3390/ijerph18158096)
Supplement: Supplementary file 1 [file ijerph-18-08096-s001.zip › Table S10.pdf]

Table S10. Baseline characteristics of study participants according to each time orientation (Female)

Variables	Year		Month		Date		Day of the week		Season	
	Wrong (n=176)	Right (n=1227)	Wrong (n=31)	Right (n=1372)	Wrong (n=97)	Right (n=1306)	Wrong (n=62)	Right (n=1341)	Wrong (n=26)	Right (n=1377)
Age, years	77.3±3.9	75.4±3.8†	79.1±4.2	75.6±3.8†	78.1±3.9	75.5±3.8†	77.3±4.1	75.6±3.8*	77.6±3.7	75.6±3.8*
BMI	24.7±3.1	24.9±3.0	24.6±3.6	24.9±3.0	24.5±3.4	24.9±3.0	24.8±3.6	24.9±3.0	25.6±3.6	24.9±3.0
Polypharmacy	68 (38.6)	338 (27.5)*	13 (41.9)	393 (28.6)	32 (33.0)	374 (28.6)	22 (35.5)	384 (28.6)	5 (19.2)	401 (29.1)
Smoking	12 (6.8)	24 (2.0)†	1 (3.2)	35 (2.6)	3 (3.1)	33 (2.5)	2 (3.2)	34 (2.5)	0 (0.0)	36 (2.6)
Alcohol drinking	8 (4.5)	46 (3.7)	1 (3.2)	53 (3.9)	3 (3.1)	51 (3.9)	1 (1.6)	53 (4.0)	1 (3.8)	53 (3.8)
Education, ≥7 years	21 (11.9)	572 (46.6)†	3 (9.7)	590 (43.0)†	17 (17.5)	576 (44.1)†	15 (24.2)	578 (43.1)*	7 (26.9)	586 (42.6)
Cell phone use	130 (73.9)	620 (50.5)†	25 (80.6)	725 (52.8)†	74 (76.3)	676 (51.8)†	43 (69.4)	707 (52.7)*	17 (65.4)	733 (53.2)*
Living alone	83 (47.2)	431 (35.1)*	10 (32.3)	504 (36.7)	46 (47.4)	468 (35.8)*	24 (38.7)	490 (36.5)	8 (30.8)	506 (36.7)
Urban	103 (58.5)	945 (77.6)†	19 (61.3)	1029 (75.5)	47 (48.5)	1001 (77.2)†	42 (68.9)	1006 (75.5)	16 (61.5)	1032 (75.4)
Medical aid	9 (5.3)	67 (5.6)	2 (7.1)	74 (5.5)	7 (7.5)	69 (5.4)	5 (8.1)	71 (5.4)	0 (0.0)	76 (5.6)
Hypertension	121 (68.8)	730 (59.5)*	19 (61.3)	832 (60.6)	53 (54.6)	798 (61.1)	46 (74.2)	805 (60.0)*	17 (65.4)	834 (60.6)
Dyslipidemia	56 (31.8)	516 (42.1)*	7 (22.6)	565 (41.2)*	34 (35.1)	538 (41.2)	20 (32.3)	552 (41.2)	6 (23.1)	566 (41.1)
Angina	11 (6.3)	65 (5.3)	0 (0.0)	76 (5.5)	5 (5.2)	71 (5.4)	8 (12.9)	68 (5.1)*	1 (3.8)	75 (5.4)
Osteoarthritis	70 (39.8)	434 (35.4)	11 (35.5)	493 (35.9)	39 (40.2)	465 (35.6)	23 (37.1)	481 (35.9)	7 (26.9)	497 (36.1)
Diabetes mellitus	32 (18.2)	251 (20.5)	7 (22.6)	276 (20.1)	25 (25.8)	258 (19.8)	10 (16.1)	273 (20.4)	6 (23.1)	277 (20.1)
Kidney disease	0 (0.0)	18 (1.5)	0 (0.0)	18 (1.3)	0 (0.0)	18 (1.4)	0 (0.0)	18 (1.3)	0 (0.0)	18 (1.3)
MCI	80 (45.5)	259 (21.1)†	15 (48.4)	324 (23.6)*	43 (44.3)	296 (22.7)†	26 (41.9)	313 (23.3)*	10 (38.5)	329 (23.9)
MMSE, score	20.7±3.7	25.9±2.7†	17.8±4.2	25.4±3.1†	20.8±4.1	25.6±3.0†	20.6±4.2	25.4±3.1†	19.4±5.1	25.3±3.2†
TMT, s	168.0±95.8	85.9±59.3†	178.7±113.9	94.3±68.1†	160.1±92.6	91.4±66.1†	158.0±100.7	93.3±67.4†	137.1±80.4	95.4±70.1*

Digit span backward, score	2.3±1.3	3.3±1.0†	2.4±1.4	3.2±1.1†	2.4±1.1	3.2±1.1†	2.6±1.3	3.2±1.1†	2.7±1.5	3.2±1.1
FAB, score	10.7±2.9	13.4±2.8†	10.2±2.6	13.1±2.9†	10.6±2.9	13.2±2.9†	11.1±3.1	13.1±2.9†	10.3±3.4	13.1±2.9†
Word list recall, score	3.7±2.0	5.9±2.0†	3.1±2.0	5.7±2.1†	3.8±2.2	5.8±2.0†	4.0±2.6	5.7±2.0†	4.5±2.4	5.6±2.1*

All values are presented as mean ± standard deviation or number (%). Depression was defined as a GDS score ≥6. Polypharmacy was defined as taking five or more prescribed medications. Alcohol consumption was defined as ≥2 or 3 or more alcoholic drinks per week. Smoking was defined as lifetime consumption of ≥5 packs of cigarettes. Education was defined as lifetime education period of ≥7 years. MMSE, Mini-Mental State Examination; TMT, trail-making test (out of 360 s); digit span backward (total score of 8); FAB, frontal assessment battery (total score of 18); recall test (total score of 10); GDS, geriatric depression scale (range 0 to 15, higher scores represent more severe depression). *$p<0.05$; †$p<0.001$.
