# Supplementary material for: Usefulness of Orientation to the Year as an Aid to Case Finding of Mild Cognitive Impairment or Depression in Community-Dwelling Older Adults
Source: Int J Environ Res Public Health. 2021 Jul 30;18(15):8096. doi: 10.3390/ijerph18158096 (PMC8345456; doi:10.3390/ijerph18158096)
Supplement: Supplementary file 1 [file ijerph-18-08096-s001.zip › Table S11.pdf]

Table S11. Time orientation tests for the diagnosis of MCI (Female)

	Sensitivity	Specificity	PPV	NPV	Accuracy
Year (wrong)	23.6%	91.0%	45.5%	78.9%	74.7%
Month (wrong)	4.4%	98.5%	48.4%	76.4%	75.8%
Date (wrong)	12.7%	94.9%	44.3%	77.3%	75.1%
Day of the week (wrong)	7.7%	96.6%	41.9%	76.7%	75.1%
Season (wrong)	2.9%	98.5%	38.5%	76.1%	75.4%

PPV, positive predictive value; NPV, negative predictive value; Accuracy, proportion of true results among the total number of cases examined.
