# Supplementary material for: Usefulness of Orientation to the Year as an Aid to Case Finding of Mild Cognitive Impairment or Depression in Community-Dwelling Older Adults
Source: Int J Environ Res Public Health. 2021 Jul 30;18(15):8096. doi: 10.3390/ijerph18158096 (PMC8345456; doi:10.3390/ijerph18158096)
Supplement: Supplementary file 1 [file ijerph-18-08096-s001.zip › Table S12.pdf]

Table S12. Time orientation tests for the diagnosis of depression (GDS score ≥6) (Female)

	Sensitivity	Specificity	PPV	NPV	Accuracy
Year (wrong)	19.3%	90.0%	42.6%	74.5%	70.5%
Month (wrong)	3.6%	98.3%	45.2%	72.7%	72.1%
Date (wrong)	9.3%	94.0%	37.1%	73.0%	70.6%
Day of the week (wrong)	4.9%	95.8%	30.6%	72.5%	70.6%
Season (wrong)	3.1%	98.6%	46.2%	72.7%	72.2%

GDS, geriatric depression scale (range 0 to 15, higher scores represent more severe depression).
