# Supplementary material for: Usefulness of Orientation to the Year as an Aid to Case Finding of Mild Cognitive Impairment or Depression in Community-Dwelling Older Adults
Source: Int J Environ Res Public Health. 2021 Jul 30;18(15):8096. doi: 10.3390/ijerph18158096 (PMC8345456; doi:10.3390/ijerph18158096)
Supplement: Supplementary file 1 [file ijerph-18-08096-s001.zip › Table S13.pdf]

Table S13. Time orientation tests for the diagnosis of MCI or depression (Female)

	Sensitivity	Specificity	PPV	NPV	Accuracy
Year (wrong)	19.8%	93.0%	68.8%	60.0%	61.1%
Month (wrong)	3.4%	98.7%	67.7%	56.9%	57.2%
Date (wrong)	10.1%	95.6%	63.9%	57.9%	58.3%
Day of the week (wrong)	6.0%	96.8%	59.7%	57.1%	57.2%
Season (wrong)	2.8%	98.9%	65.4%	56.8%	56.9%
