# Supplementary material for: Usefulness of Orientation to the Year as an Aid to Case Finding of Mild Cognitive Impairment or Depression in Community-Dwelling Older Adults
Source: Int J Environ Res Public Health. 2021 Jul 30;18(15):8096. doi: 10.3390/ijerph18158096 (PMC8345456; doi:10.3390/ijerph18158096)
Supplement: Supplementary file 1 [file ijerph-18-08096-s001.zip › Table S2.pdf]

Table S2. Number of errors in three-item recall (tree, car, hat) tests for the diagnosis of depression (GDS score≥6)

Number of errors	Sensitivity	Specificity	PPV	NPV	Accuracy	Youden's index
1	65.2%	39.1%	22.7%	80.4%		0.043
2	32.5%	73.5%	25.2%	79.9%	64.7%	0.060
3	13.7%	89.5%	26.5%	79.1%		0.033

GDS, geriatric depression scale (range 0 to 15, higher scores represent more severe depression).
