# Supplementary material for: Usefulness of Orientation to the Year as an Aid to Case Finding of Mild Cognitive Impairment or Depression in Community-Dwelling Older Adults
Source: Int J Environ Res Public Health. 2021 Jul 30;18(15):8096. doi: 10.3390/ijerph18158096 (PMC8345456; doi:10.3390/ijerph18158096)
Supplement: Supplementary file 1 [file ijerph-18-08096-s001.zip › Table S3.pdf]

Table S3. Number of errors in three-item recall (tree, car, hat) tests for the diagnosis of MCI or depression

Number of errors	Sensitivity	Specificity	PPV	NPV	Accuracy	Youden's index
1	67.1%	41.3%	40.5%	67.9%		0.084
2	34.6%	76.2%	46.4%	66.2%	60.7%	0.108
3	15.1%	91.2%	50.3%	64.3%		0.062
