# Supplementary material for: Usefulness of Orientation to the Year as an Aid to Case Finding of Mild Cognitive Impairment or Depression in Community-Dwelling Older Adults
Source: Int J Environ Res Public Health. 2021 Jul 30;18(15):8096. doi: 10.3390/ijerph18158096 (PMC8345456; doi:10.3390/ijerph18158096)
Supplement: Supplementary file 1 [file ijerph-18-08096-s001.zip › Table S4.pdf]

Table S4. Baseline characteristics of the study subjects according to mild cognitive impairment or depression (Male)

Variable	Mild cognitive impairment		p	Depression		p	Total (N=1265)
	Non-MCI (n=1012)	MCI (n=253)		Non-depression (n=1078)	Depression (n=187)		
Age, years	76.2±3.9	76.8±3.8	0.028	76.2±3.9	77.1±3.9	0.004	76.3±3.9
BMI	24.0±2.9	23.7±2.8	0.055	24.1±2.9	23.4±2.9	0.006	24.0±2.9
Polypharmacy	347 (34.3)	95 (37.5)	0.331	364 (33.8)	78 (41.7)	0.035	442 (34.9)
Smoking	794 (78.5)	193 (76.3)	0.455	831 (77.1)	156 (83.4)	0.053	987 (78.0)
Alcohol drinking	348 (34.4)	76 (30.0)	0.190	366 (34.0)	58 (31.0)	0.432	424 (33.5)
Education, ≥7 years	752 (74.3)	185 (73.1)	0.700	830 (77.0)	107 (57.2)	<0.001	937 (74.1)
Cell phone use	410 (40.5)	139 (54.9)	<0.001	432 (40.1)	117 (62.6)	<0.001	549 (43.4)
Living alone	81 (8.0)	37 (14.6)	0.001	78 (7.2)	40 (21.4)	<0.001	118 (9.3)
Urban	711 (70.5)	148 (59.2)	0.001	739 (68.9)	120 (64.2)	0.197	859 (68.2)
Medical aid	43 (4.3)	11 (4.5)	0.891	38 (3.6)	16 (8.8)	0.001	54 (4.3)
Hypertension	544 (53.8)	134 (53.0)	0.822	577 (53.5)	101 (54.0)	0.902	678 (53.6)
Dyslipidemia	258 (25.5)	48 (19.0)	0.030	267 (24.8)	39 (20.7)	0.249	306 (24.2)
Angina	73 (7.2)	14 (5.5)	0.345	72 (6.7)	15 (8.0)	0.503	87 (6.9)
Osteoarthritis	136 (13.4)	38 (15.0)	0.514	136 (12.6)	38 (20.3)	0.005	174 (13.8)
Diabetes mellitus	237 (23.4)	59 (23.3)	0.974	251 (23.3)	45 (24.1)	0.816	296 (23.4)
Kidney disease	20 (2.0)	1 (0.4)	0.078	16 (1.5)	5 (2.7)	0.240	21 (1.7)
Time orientation							
Year, wrong	29 (2.9)	25 (9.9)	<0.001	37 (3.4)	17 (9.1)	<0.001	54 (4.3)
Month, wrong	10 (1.0)	6 (2.4)	0.078	11 (1.0)	5 (2.7)	0.062	16 (1.3)
Date, wrong	24 (2.4)	19 (7.5)	<0.001	33 (3.1)	10 (5.3)	0.111	43 (3.4)
Day of the week, wrong	57 (5.6)	33 (13.0)	<0.001	78 (7.2)	12 (6.4)	0.688	90 (7.1)
Season, wrong	12 (1.2)	14 (5.5)	<0.001	25 (2.3)	1 (0.5)	0.112	26 (2.1)
MMSE, score	26.7±2.6	24.6±3.4	<0.001	26.4±2.8	25.3±3.1	<0.001	26.3±2.9
TMT, s	58.2±26.2	90.1±62.1	<0.001	62.4±38.3	76.9±36.9	<0.001	64.6±38.5
Digit span backward, score	3.8±0.9	2.7±1.1	<0.001	3.6±1.0	3.3±1.0	<0.001	3.6±1.0

FAB, score	14.8±2.1	12.0±3.3	<0.001	14.4±2.6	13.0±2.8	<0.001	14.2±2.6
Word list recall, score	5.9±1.8	4.2±2.2	<0.001	5.7±2.0	4.7±2.1	<0.001	5.5±2.0

All values are presented as mean ± standard deviation or number (%).Depression was defined as a GDS score ≥6. Polypharmacy was defined as taking five or more prescribed medications. Alcohol consumption was defined as ≥2 or 3 or more alcoholic drinks per week. Smoking was defined as lifetime consumption of ≥5 packs of cigarettes. Education was defined as lifetime education period of ≥7 years. MMSE, Mini-Mental State Examination; TMT, trail-making test (out of 360 s); digit span backward (total score of 8); FAB, frontal assessment battery (total score of 18); recall test (total score of 10); GDS, geriatric depression scale (range 0 to 15, higher scores represent more severe depression).
