# Supplementary material for: Usefulness of Orientation to the Year as an Aid to Case Finding of Mild Cognitive Impairment or Depression in Community-Dwelling Older Adults
Source: Int J Environ Res Public Health. 2021 Jul 30;18(15):8096. doi: 10.3390/ijerph18158096 (PMC8345456; doi:10.3390/ijerph18158096)
Supplement: Supplementary file 1 [file ijerph-18-08096-s001.zip › Table S5.pdf]

Table S5. Baseline characteristics of study participants according to each time orientation (Male)

Variables	Year		Month		Date		Day of the week		Season	
	Wrong (n=54)	Right (n=1211)	Wrong (n=16)	Right (n=1249)	Wrong (n=43)	Right (n=1222)	Wrong (n=90)	Right (n=1175)	Wrong (n=26)	Right (n=1239)
Age, years	77.6±4.2	76.2±3.9*	77.4±3.2	76.3±3.9	77.7±3.6	76.2±3.9*	77.5±4.2	76.2±3.8*	77.8±4.4	76.3±3.9*
BMI	23.4±3.3	24.0±2.9	23.7±2.7	24.0±2.9	23.9±2.8	24.0±2.9	23.5±2.9	24.0±2.9	22.7±3.4	24.0±2.9*
Polypharmacy	23 (42.6)	419 (34.6)	5 (31.3)	437 (35.0)	17 (39.5)	425 (34.8)	33 (36.7)	409 (34.8)	10 (38.5)	432 (34.9)
Smoking	45 (83.3)	942 (77.8)	12 (75.0)	975 (78.1)	35 (81.4)	952 (77.9)	71 (78.9)	916 (78.0)	16 (61.5)	971 (78.4)*
Alcohol drinking	18 (33.3)	406 (33.5)	4 (25.0)	420 (33.6)	16 (37.2)	408 (33.4)	22 (24.4)	402 (34.2)	13 (50.0)	411 (33.2)
Education, ≥7 years	27 (50.0)	910 (75.1)†	10 (62.5)	927 (74.2)	31 (72.1)	906 (74.1)	52 (57.8)	885 (75.3)†	15 (57.7)	922 (74.4)
Cell phone use	14 (25.9)	690 (57.0)†	9 (56.3)	540 (43.2)*	22 (51.2)	527 (43.1)	50 (55.6)	499 (42.5)*	15 (57.7)	534 (43.1)†
Living alone	6 (11.1)	112 (9.2)	3 (18.8)	115 (9.2)	7 (16.3)	111 (9.1)	13 (14.4)	105 (8.9)	3 (11.5)	115 (9.3)
Urban	29 (53.7)	830 (68.9)*	11 (68.8)	848 (68.2)	33 (76.7)	826 (67.9)	51 (57.3)	808 (69.1)*	12 (48.0)	847 (68.6)*
Medical aid	3 (5.6)	51 (4.3)	0 (0.0)	54 (4.4)	3 (7.1)	51 (4.2)	6 (6.8)	48 (4.1)	0 (0.0)	54 (4.4)
Hypertension	24 (44.4)	654 (54.0)	9 (56.3)	669 (53.6)	27 (62.8)	651 (53.3)	45 (50.0)	633 (53.9)	11 (42.3)	667 (53.8)
Dyslipidemia	11 (20.4)	295 (24.4)	4 (25.0)	302 (24.2)	14 (32.6)	292 (23.9)	20 (22.2)	286 (24.3)	10 (38.5)	296 (23.9)
Angina	5 (9.3)	82 (6.8)	0 (0.0)	87 (7.0)	3 (7.0)	84 (6.9)	5 (5.6)	82 (7.0)	1 (3.8)	86 (6.9)
Osteoarthritis	8 (14.8)	166 (13.7)	5 (31.3)	169 (13.5)*	4 (9.3)	170 (13.9)	18 (20.0)	156 (13.3)	7 (26.9)	167 (13.5)*
Diabetes mellitus	10 (18.5)	286 (23.6)	4 (25.0)	292 (23.4)	13 (30.2)	283 (23.2)	24 (26.7)	272 (23.1)	8 (30.8)	288 (23.2)
Kidney disease	3 (5.6)	18 (1.5)*	0 (0.0)	21 (1.7)	0 (0.0)	21 (1.7)	2 (2.2)	19 (1.6)	0 (0.0)	21 (1.7)
MCI	25 (46.3)	228 (18.8)†	6 (37.5)	247 (19.8)	19 (44.2)	234 (19.1)†	33 (36.7)	220 (18.7)†	14 (53.8)	239 (19.3)†
MMSE, score	21.2±4.6	26.5±2.5†	20.8±5.3	26.4±2.8*	23.0±4.1	26.4±2.8†	23.5±3.9	26.5±2.7†	21.8±5.6	26.4±2.7†
TMT, s	106.5±74.0	62.7±35.0†	86.8±37.9	64.3±38.4*	81.7±61.6	64.0±37.3	85.8±54.6	62.9±36.5†	112.2±91.5	63.6±36.0*

Digit span backward, score	2.9±1.3	3.6±1.0†	2.8±1.4	3.6±1.0*	3.5±1.2	3.6±1.0	3.3±1.3	3.6±1.0*	3.1±1.8	3.6±1.0
FAB, score	11.6±3.3	14.3±2.5†	12.3±3.4	14.3±2.6*	12.7±2.8	14.3±2.6†	12.5±3.1	14.4±2.6†	12.2±4.1	14.3±2.6*
Word list recall, score	3.4±2.1	5.6±2.0†	4.3±2.4	5.5±2.0*	3.8±2.2	5.6±2.0†	4.1±2.0	5.6±2.0†	4.3±2.0	5.5±2.0*

All values are presented as mean ± standard deviation or number (%). Depression was defined as a GDS score ≥6. Polypharmacy was defined as taking five or more prescribed medications. Alcohol consumption was defined as ≥2 or 3 or more alcoholic drinks per week. Smoking was defined as lifetime consumption of ≥5 packs of cigarettes. Education was defined as lifetime education period of ≥7 years. MMSE, Mini-Mental State Examination; TMT, trail-making test (out of 360 s); digit span backward (total score of 8); FAB, frontal assessment battery (total score of 18); recall test (total score of 10); GDS, geriatric depression scale (range 0 to 15, higher scores represent more severe depression). *$p<0.05$; †$p<0.001$.
