# Supplementary material for: Usefulness of Orientation to the Year as an Aid to Case Finding of Mild Cognitive Impairment or Depression in Community-Dwelling Older Adults
Source: Int J Environ Res Public Health. 2021 Jul 30;18(15):8096. doi: 10.3390/ijerph18158096 (PMC8345456; doi:10.3390/ijerph18158096)
Supplement: Supplementary file 1 [file ijerph-18-08096-s001.zip › Table S6.pdf]

Table S6. Time orientation tests for the diagnosis of MCI (Male)

	Sensitivity	Specificity	PPV	NPV	Accuracy
Year (wrong)	9.9%	97.1%	46.3%	81.2%	79.7%
Month (wrong)	2.4%	99.0%	37.5%	80.2%	79.7%
Date (wrong)	7.5%	97.6%	44.2%	80.9%	79.6%
Day of the week (wrong)	13.0%	94.4%	36.7%	81.3%	78.1%
Season (wrong)	5.5%	98.8%	53.8%	80.7%	80.2%

PPV, positive predictive value; NPV, negative predictive value; Accuracy, proportion of true results among the total number of cases examined.
