# Supplementary material for: Usefulness of Orientation to the Year as an Aid to Case Finding of Mild Cognitive Impairment or Depression in Community-Dwelling Older Adults
Source: Int J Environ Res Public Health. 2021 Jul 30;18(15):8096. doi: 10.3390/ijerph18158096 (PMC8345456; doi:10.3390/ijerph18158096)
Supplement: Supplementary file 1 [file ijerph-18-08096-s001.zip › Table S7.pdf]

Table S7. Time orientation tests for the diagnosis of depression (GDS score ≥6) (Male)

	Sensitivity	Specificity	PPV	NPV	Accuracy
Year (wrong)	9.1%	96.6%	31.5%	86.0%	83.6%
Month (wrong)	2.7%	99.0%	31.3%	85.4%	84.7%
Date (wrong)	5.3%	96.9%	23.3%	85.5%	83.4%
Day of the week (wrong)	6.4%	92.8%	13.3%	85.1%	80.0%
Season (wrong)	0.5%	97.7%	3.8%	85.0%	83.3%

GDS, geriatric depression scale (range 0 to 15, higher scores represent more severe depression).
