# Supplementary material for: Usefulness of Orientation to the Year as an Aid to Case Finding of Mild Cognitive Impairment or Depression in Community-Dwelling Older Adults
Source: Int J Environ Res Public Health. 2021 Jul 30;18(15):8096. doi: 10.3390/ijerph18158096 (PMC8345456; doi:10.3390/ijerph18158096)
Supplement: Supplementary file 1 [file ijerph-18-08096-s001.zip › Table S8.pdf]

Table S8. Time orientation tests for the diagnosis of MCI or depression (Male)

	Sensitivity	Specificity	PPV	NPV	Accuracy
Year (wrong)	8.6%	97.6%	61.1%	71.1%	70.7%
Month (wrong)	2.3%	99.2%	56.3%	70.1%	69.9%
Date (wrong)	6.5%	98.0%	58.1%	70.7%	70.3%
Day of the week (wrong)	10.7%	94.4%	45.6%	70.9%	69.1%
Season (wrong)	3.7%	98.6%	53.8%	70.2%	69.9%
