# Supplementary material for: Usefulness of Orientation to the Year as an Aid to Case Finding of Mild Cognitive Impairment or Depression in Community-Dwelling Older Adults
Source: Int J Environ Res Public Health. 2021 Jul 30;18(15):8096. doi: 10.3390/ijerph18158096 (PMC8345456; doi:10.3390/ijerph18158096)
Supplement: Supplementary file 1 [file ijerph-18-08096-s001.zip › Table S9.pdf]

Table S9. Baseline characteristics of the study subjects according to mild cognitive impairment or depression (Female)

Variable	Mild cognitive impairment		p	Depression		p	Total (N=1403)
	Non-MCI (n=1064)	MCI (n=339)		Non-depression (n=1015)	Depression (n=388)		
Age, years	76.5±3.8	75.4±3.8	<0.001	76.4±3.9	75.3±3.8	<0.001	75.6±3.9
BMI	24.9±3.0	24.9±3.0	0.929	24.9±3.1	24.9±3.0	0.889	24.9±3.0
Polypharmacy	292 (27.4)	114 (33.6)	0.029	266 (26.2)	140 (36.1)	<0.001	406 (28.9)
Smoking	21 (2.0)	15 (4.4)	0.013	21 (2.1)	15 (3.9)	0.057	36 (2.6)
Alcohol drinking	42 (3.9)	12 (3.5)	0.734	40 (3.9)	14 (3.6)	0.772	54 (3.8)
Education, ≥7 years	499 (46.9)	94 (27.7)	<0.001	475 (46.8)	118 (30.4)	<0.001	593 (42.3)
Cell phone use	528 (49.6)	222 (65.5)	<0.001	485 (47.8)	265 (68.3)	<0.001	750 (53.5)
Living alone	368 (34.6)	146 (43.1)	0.005	339 (33.4)	175 (45.1)	<0.001	514 (36.6)
Urban	826 (78.2)	222 (65.7)	<0.001	767 (76.1)	281 (72.8)	0.203	1048 (75.2)
Medical aid	59 (5.6)	17 (5.2)	0.752	41 (4.1)	35 (9.3)	<0.001	76 (5.5)
Hypertension	634 (59.6)	217 (64.0)	0.146	616 (60.7)	235 (60.6)	0.966	851 (60.7)
Dyslipidemia	461 (43.3)	111 (32.7)	0.001	417 (41.1)	155 (39.9)	0.699	572 (40.8)
Angina	58 (5.5)	18 (5.3)	0.920	55 (5.4)	21 (5.4)	0.996	76 (5.4)
Osteoarthritis	375 (35.2)	129 (38.1)	0.348	353 (34.8)	151 (38.9)	0.148	504 (35.9)
Diabetes mellitus	210 (19.7)	73 (21.5)	0.473	207 (20.4)	76 (19.6)	0.736	283 (20.2)
Kidney disease	15 (1.4)	3 (0.9)	0.455	11 (1.1)	7 (1.8)	0.284	18 (1.3)
Time orientation							
Year, wrong	96 (9.0)	80 (23.6)	<0.001	101 (10.0)	75 (19.3)	<0.001	176 (12.5)
Month, wrong	16 (1.5)	15 (4.4)	0.001	17 (1.7)	14 (3.6)	0.028	31 (2.2)
Date, wrong	54 (5.1)	43 (12.7)	<0.001	61 (6.0)	36 (9.3)	0.031	97 (6.9)
Day of the week, wrong	36 (3.4)	26 (7.7)	0.001	43 (4.2)	19 (4.9)	0.590	62 (4.4)
Season, wrong	16 (1.5)	10 (2.9)	0.086	14 (1.4)	12 (3.1)	0.033	26 (1.9)
MMSE, score	23.5±3.9	25.8±3.0	<0.001	24.2±3.6	25.6±3.2	<0.001	25.2±3.3
TMT, s	146.6±99.0	80.1±48.6	<0.001	119.4±79.7	87.3±64.4	<0.001	96.2±70.4
Digit span backward, score	2.2±1.1	3.5±0.9	<0.001	2.8±1.1	3.3±1.1	<0.001	3.2±1.1
FAB, score	10.6±3.0	13.8±2.5	<0.001	12.0±3.0	13.4±2.9	<0.001	13.0±3.0

Word list recall, score	4.5±2.4	6.0±1.9	<0.001	5.2±2.1	5.8±2.1	<0.001	5.6±2.1

All values are presented as mean ± standard deviation or number (%).Depression was defined as a GDS score ≥6. Polypharmacy was defined as taking five or more prescribed medications. Alcohol consumption was defined as ≥2 or 3 or more alcoholic drinks per week. Smoking was defined as lifetime consumption of ≥5 packs of cigarettes. Education was defined as lifetime education period of ≥7 years. MMSE, Mini-Mental State Examination; TMT, trail-making test (out of 360 s); digit span backward (total score of 8); FAB, frontal assessment battery (total score of 18); recall test (total score of 10); GDS, geriatric depression scale (range 0 to 15, higher scores represent more severe depression).
